# The Short Cut to Sanity

**Published:** 1921-04-23

**Authors:** 


### THE SHORT CUT TO SANITY.

It is remarkable how blind mankind is to the obvious. How many people before Newton had seen—or felt—an apple drop. And yet, fortunately, there are always the enlightened few who can lead us towards the region of clearer vision. Consider how we have laboriously struggled to elucidate the many problems of nervous disorders, blind all the while to the few simple propositions which would unfold for us the tangled skein! The records of the past do, however, justify some pessimism as to the possibility of such simplification of our difficulties as some enthusiastic exponents of the new cult of psycho-analysis would have us believe. There has been similar enthusiasm about shrines and other methods of faith-healing, hypnotism, mesmerism, and about the thousand and one fashionable therapeutic cults, and usually they have been accompanied by sneering remarks as to the incompetence of the legitimate practitioners of the healing art and their impermeability to new doctrines. But these cults have had their day—more or less—and the good that was in them has had much difficulty in surviving the inevitable obloquy which is the portion of those who fail to fulfil promises. They have failed because they were unable to realise their limitations, and thus applied their remedies to cases which were totally unsuitable. That was the inevitable result of ignorance. There were others who brought discredit upon themselves not through ignorance—though that was often enough to be discerned—but because they realised that they had an easy way to the pockets of the credulous. Of course, where ignorance and rapacity are united, *à fortiori*, the battening process will be accelerated.

These are some of the dangers with which the path of the psycho-analyst is beset. Many people have already realised this. They will feel all the more anxious when they see such a statement as that a medical education *per se* does not necessarily imply the requisite qualifications for successfully practising psycho-analysis; and that those medical practitioners who have excelled in this method have done so not necessarily "by virtue of their orthodox medical training, but in spite of it."* These statements are quoted with approval in the lay press.† Were we misanthropists we should say sardonically that those who hold such opinions should be left to themselves to await their disillusionment. But we have a duty to the public and cannot, therefore, stand thus aside. The law, too, has obligations which it must not neglect. Legislative enactments were made for the protection of the public so that it should not be at the mercy of anyone who might, without producing any credentials, prey upon the community. Instead of these rules being relaxed they should be made even more stringent; and not even medical men should be allowed to practise such a method of treatment without having previously acquired a competent knowledge of mental disorders and of neurology. This would obviate the scandal of progressive organic disease, for example, general paralysis of the insane, being treated by "psychological" methods.

It is idle to say that the medical profession is not alert for advances in therapeutics. It may be true of some of the ultra-conservatives; there are, however, always many medical men alive to scientific progress, indeed, it is they who serve to carry forward the lamp in spite of criticism and even of detraction. But it is their duty to see that misguided enthusiasm and self-seeking charlatanism are not allowed to inflict injury upon the body politic. They must not be deterred from making use of that which is good in such a method as psycho-analysis by the misguided optimism of the uninstructed. Within limits this method has its utility; but the people best qualified to define those limits are not the enthusiastic amateurs who, devoid of experience and of the cautiousness such experience would bring, imagine that they have discovered a short cut to sanity. In any case it is well for us all to remember that what we should bear steadily in mind is the need for cutting off as much mental disorder as we can at its source by means of prophylactic measures. But that is another story.

* "The Psychology of Nervous Ailments." A Brief Description of the Freudian Analytic Method of Adjusting Nervous Disturbances. By Joseph Ralph.

† *The Bookman's Journal*, March 25, 1921, p. 394.

